# Chronic kidney disease and diabetes associated with long-term outcomes in patients receiving percutaneous coronary intervention

**DOI:** 10.1186/s12872-017-0673-4

**Published:** 2017-09-11

**Authors:** Mao-Jen Lin, Jung Lee, Chun-Yu Chen, Chia-Chen Huang, Han-Ping Wu

**Affiliations:** 1 Division of Cardiology, Department of Medicine, Taichung Tzu Chi Hospital, The Buddhist Tzu Chi Medical foundation, Taichung, Taiwan; 2 Department of Medicine, School of Medicine, Tzu Chi University, Hualien, Taiwan; 3 Division of Pediatric General Medicine, Department of Pediatrics, Chang Gung Memorial Hospital at Linko, No. 5, Fu-Hsin Street, Kweishan, Taoyuan Taiwan; 4 College of Medicine, Chang Gung University, Taoyuan, Taiwan; 5 Department of Pediatric Emergency Medicine, Changhua Christian Children’s Hospital, Changhua, Taiwan; 6 School of Medicine, Kaohsiung Medical University, Kaohsiung, Taiwan; 7 Department of Public Health, Chung Shan Medical University, Taichung, Taiwan

**Keywords:** PCI, Diabetes mellitus, Chronic kidney disease

## Abstract

**Background:**

The effect of diabetes mellitus (DM) and chronic kidney disease (CKD) on long-term outcomes in patients receiving percutaneous coronary intervention (PCI) is unclear.

**Methods:**

A total of 1394 patients who underwent PCI were prospectively enrolled and divided into 4 groups according to the presence or absence of DM or CKD. Baseline characteristics, risk factors, medications, and angiographic findings were compared. Determinants of long-term outcomes in patients undergoing PCI were analyzed.

**Results:**

Patients with DM and CKD had the highest all-cause mortality and cardiovascular mortality (both *P* < 0.01) but there were no differences existed in myocardial infarction (MI) or repeated PCI among the 4 groups (*P* = 0.19, *P* = 0.87, respectively). Patients with DM and CKD had the lowest even-free rate of all-cause mortality, cardiovascular mortality, MI, and repeated PCI (*P* < 0.001, *P* < 0.001, *P* < 0.001, and *P* = 0.002, respectively). In the Cox proportional hazard model, patients with both DM and CKD had the highest risk of all-cause mortality (HR: 3.25, 95% CI: 1.85–5.59), cardiovascular mortality (HR: 3.58, 95% CI: 1.97–6.49), MI (HR: 2.43, 95% CI: 1.23–4.08), and repeated PCI (HR: 1.79, 95% CI: 1.33–2.41). Patients with CKD alone had the second highest risk of all-cause mortality (HR: 2.04, 95% CI: 1.15–3.63), cardiovascular mortality (HR: 2.13, 95% CI: 1.13–4.01), and repeated PCI (HR: 1.47, 95% CI: 1.09–1.97).

**Conclusions:**

DM and CKD had additive effect on adverse long-term outcomes in patients receiving PCI; CKD was a more significant adverse predictor than DM.

## Background

Percutaneous coronary intervention (PCI) is common in patients with coronary artery disease(CAD). However, a lot of risk factors will affect the outcome after patients receiving PCI. Diabetes mellitus (DM) is a major risk factor that affects outcomes in CAD patients undergoing PCI [1–3]. Recently, chronic kidney disease (CKD) has emerged as a risk factor in terms of outcomes in patients undergoing PCI [4–7].

The impact of DM and CKD on outcomes has been well studied in patients with acute coronary syndrome (ACS) undergoing PCI. Presence of CKD could affect long-term outcomes in patients with ST-elevation myocardial infarction (STEMI) managed by primary PCI and in-hospital mortality in patients with non-ST elevation myocardial infarction (non-STEMI) undergoing PCI [8, 9]. After undergoing PCI, diabetic patients suffered from ACS had worse short-term and mid-term outcomes than non-diabetic patients with ACS [10–12]. As for patients with stable CAD who underwent PCI, diabetes was still an adverse predictor of mid-term outcomes.

For ACS patients with both DM and CKD, the coexistence of DM and CKD appeared to have the higher risk of MACE than DM alone or CKD alone [13]. However, the combined effect of DM and CKD on long-term prognosis in patients undergoing PCI is still unclear. Our aim was to clarify and compare long-term outcomes among four groups of patients: patients with both DM and CKD, with only DM, with only CKD, and without CKD and DM. We further analyzed the adverse predictors of clinical outcomes among these 4 groups.

## Methods

### Study population

A prospective cohort design using was conducted via medical records review from May 2007 to Dec 2014. The institutional review board and ethics committee approved the study protocol and monitoring for the study.

We consecutively recruited patients aged 30 to 90 years who were to undergo PCI from the inpatient clinic at the Taichung Tzu Chi Hospital, Taiwan. According to isolated or coexistence of risk factors, the patients were divided into four groups: patients without DM or CKD, patients with DM alone, patients with CKD alone, and patients with both DM and CKD. Patients with a scheduled PCI and previous history of malignancy were excluded. Most patients obtained regular follow-up in the outpatient department (OPD). For some patients who were lost to follow-up, usually a phone call was used to contact the patients themselves or their families. A survey of cardiovascular mortality (CV mortality), all-cause mortality, myocardial infarction (MI), and repeated PCI procedures was completed at the end of the study.

### Data gathering and analysis

Baseline characteristics including body habitus, biochemical data, angiographic findings from cardiac catheterization, exposed risk factors, and variant therapeutic strategies such as drug medications and invasive procedures (balloon angioplasty, bare metal stent deployment, or drug-eluting stent deployment) were all obtained for our study. Diabetes was defined as a fasting plasma glucose level of >126 mg/dL, a casual plasma glucose level of >200 mg/dL, or a hemoglobin A1c (HbA1c) level of >6.5% in this study [14]. Estimated glomerular filtration rate (eGFR) and chronic kidney disease (CKD) stage were divided into 5 stages: stage l, eGFR ≥90 mL/min/1.73 m^2^; stage 2, eGFR of 60–89 mL/min/1.73 m^2^; stage 3, eGFR of 30–59 mL/min/1.73 m^2^; stage 4, eGFR of 15–29 mL/min/1.73 m^2^; and stage 5, eGFR of <15 mL/min/1.73 m^2^ or dialysis. CKD was defined as an eGFR of <60 mL/min/1.73 m^2^, which was equal to or greater than CKD stage 3, in this study [15]. Hypercholesterolemia was defined as a serum cholesterol level of >200 mg/dL or an LDL-C level of >100 mg/dLFor the angiographic and hemodynamic data, we measured the central aortic pressure (CAP) and left ventricular ejection fraction (LVEF). The CAP was obtained by using a pigtail catheter while performing a coronary angiography. Angiographic findings including number of diseased vessels and lesion locations were calculated, and lesion severity and complexity were evaluated via the Synergy between PCI with Taxus and cardiac surgery score (Syntax Score) [16]. The left ventricular ejection was estimated through angiographic ventriculography or stress ventriculography.MI was defined as an MI attack after index PCI, accompanied by a 3-fold elevation in cardiac enzymes from the baseline value. General characteristics, major risk factors, angiographic findings, PCI strategies were analyzed, The primary end-points including all-cause death, cardiovascular death, myocardial infarction and repeated PCI were also compared among the 4 groups. The beginning of follow-up was the date of index PCI, and the duration of follow up was from the beginning through June 30, 2015 or if any of above primary end-points happened.

### Statistical analysis

The analysis was primarily used to assess differences among the 4 groups. Analysis of variance (ANOVA) was used for test continuous variables, and chi-square test was used to test categorical variables. Log-rank test and Kaplan–Meier curves were used to compare survival differences. Cox proportional hazards model was used to test the effect of independent variables on hazards. *P* values of <0.05 were considered significant. All analyses were performed using the statistical package SPSS for Windows (Version 23.0 SPSS Inc., Chicago, IL, USA).

## Results

During the whole study period, a total of 1394 patients who underwent a successful PCI procedure were enrolled. Among them, 509 patients in the control group had neither DM nor CKD, 254 patients had DM alone, 320 patients had CKD alone, and 311 patients had both DM and CKD. There were no differences in mean follow up time among the 4 groups (control group, 31.7 ± 22.6 months; DM alone, 31.1 ± 22.4 months; CKD alone, 23.8 ± 19.7 months; and both DM and CKD, 23.0 ± 19.2 months; *P* = 0.08).

General characteristics are listed in Table 1. Patients with CKD alone and with both DM and CKD were much older than patients in the other groups (*P <* 0.01). As for body habitus parameters, patients with CKD alone and patients with both DM and CKD had a lower body mass index(BMI), as compared with patients in the other 2 groups (*P* < 0.01). As for the hemodynamic parameters, patients with both DM and CKD had the highest central systolic pressure (CSP) and lowest central diastolic pressure (CDP), as compared with patients in the other groups (*P* < 0.01). As for baseline biochemistry, patients with both DM and CKD had the lowest cholesterol, low density lipoprotein-cholesterol(LDL-C) levels, and the poorest renal function (all *P* < 0.01).

**Table 1:** General Characteristics of the Study Population

Variable	Study Groups				*P* value
	Control (*N* = 509)	DM alone (*N* = 254)	CKD alone (*N* = 320)	DM and CKD (*N* = 311)	
Age (y)	58.1 ± 10.8	59.6 ± 10.3	72.2 ± 10.1	68.6 ± 10.3	<0.01
Weight (kg)	70.9 ± 11.8	72.2 ± 14.4	62.0 ± 12.0	64.6 ± 12.0	<0.01
Height (cm)	164.5 ± 8.0	163.6 ± 8.8	159.6 ± 7.9	160.2 ± 8.4	<0.01
BMI (kg/m^2^)	26.2 ± 3.6	26.9 ± 4.3	24.3 ± 3.9	25.1 ± 3.8	<0.01
CSP	133.4 ± 20.7	135.8 ± 23.0	136.5 ± 25.3	143.1 ± 26.3	<0.01
CDP	76.0 ± 12.3	73.8 ± 13.1	70.7 ± 13.1	70.3 ± 13.2	<0.01
Cholesterol (mg/dL)	184.4 ± 43.5	180.3 ± 45.4	177.0 ± 42.2	169.5 ± 46.6	<0.01
HDL (mg/dL)	39.4 ± 15.9	36.1 ± 14.7	43.2 ± 17.1	37.6 ± 15.4	<0.01
TG (mg/dL)	160.0 ± 105.0	171.5 ± 117.1	134.9 ± 94.1	161.2 ± 110.1	<0.01
LDL (mg/dL)	113.0 ± 39.7	109.6 ± 36.7	106.9 ± 36.3	99.7 ± 38.2	<0.01
Serum creatinine (mg/dL)	0.9 ± 0.2	1.0 ± 0.3	2.2 ± 2.4	3.2 ± 3.3	<0.01

*DM alone* diabetes alone, *CKD alone* chronic kidney disease alone, *DM and CKD* both DM and CKD, *BMI* body mass index, *CSP* central aortic systolic pressure, *CDP* central aortic diastolic pressure, *HDL* high-density lipoprotein cholesterol, *LDL* low-density lipoprotein cholesterol, *TG* triglyceride

Demographic data for the study population are shown on Table 2. Female and hypertension preponderance were observed in patients with DM and CKD (both *P* < 0.01); they also had the highest prevalence of stroke history (*P* < 0.01). However, patients with DM and CKD had the lowest prevalence of hypercholesterolemia and were the least likely to be current smokers (both *P* < 0.01). In terms of medication after PCI, we found that patients with CKD alone had the lowest aspirin use (*P* < 0.01) and highest diuretic use (*P* < 0.01). Patients with both DM and CKD had the lowest usage of angiotensin-converting enzyme inhibitors (ACEI) and statins (*P* = 0.02 and *P* < 0.01, respectively).

**Table 2:** Demographic Characteristics of the Study Population and Medication Use after the First PCI

Variable	Study Group				*P* value
	Control (*N* = 509)	DM Alone (*N* = 254)	CKD Alone (*N* = 320)	DM and CKD *N* = 311)	
Gender					<0.01
F	87 (17.1%)	60 (23.6%)	99 (30.9%)	120 (38.6%)	
M	422 (82.9%)	194 (76.4%)	221 (69.1%)	191 (61.4%)	
Hypertension					<0.01
No	265 (52.1%)	103 (40.6%)	120 (37.5%)	91 (29.3%)	
Yes	244 (47.9%)	151 (59.4%)	200 (62.5%)	220 (70.7%)	
Hypercholesterolemia					<0.01
No	189 (37.1%)	115 (45.3%)	139 (43.4%)	178 (57.2%)	
Yes	320 (62.9%)	139 (54.7%)	181 (56.6%)	133 (42.8%)	
Current smoker					<0.01
No	269 (52.8%)	165 (65.0%)	202 (63.3%)	237 (76.2%)	
Yes	240 (47.2%)	89 (35.0%)	118 (36.7%)	74 (23.8%)	
MI					0.07
No	349 (68.5%)	170 (66.9%)	191 (59.7%)	202 (65.0%)	
Yes	160 (31.5%)	84 (33.1%)	129 (40.3%)	109 (35.0%)	
Stroke history					<0.01
No	492 (96.7%)	242 (95.3%)	297 (92.8%)	283 (91.0%)	
Yes	17 (3.3%)	12 (4.7%)	23 (7.2%)	28 (9.0%)	
CABG history					0.06
No	509 (100.0%)	252 (99.2%)	317 (99.1%)	306 (98.4%)	
Yes	0 (0.0%)	2 (0.8%)	3 (0.9%)	5 (1.6%)	
Asprin					<0.01
No	25 (4.9%)	15 (5.9%)	43 (13.4%)	37 (11.9%)	
Yes	484 (95.1%)	239 (94.1%)	277 (86.6%)	274 (88.1%)	
P2Y12 inhibitors					0.72
No	90 (17.7%)	43 (16.9%)	47 (14.7%)	50 (16.1%)	
Yes	419 (82.3%)	211 (83.1%)	273 (85.3%)	261 (83.9%)	
Diuretics					<0.01
No	435 (85.5%)	189 (74.4%)	234 (73.1%)	229 (73.6%)	
Yes	74 (14.5%)	65 (25.6%)	86 (26.9%)	82 (26.4%)	
Beta B					0.31
No	283 (55.6%)	130 (51.2%)	189 (59.1%)	175 (56.3%)	
Yes	226 (44.4%)	124 (48.8%)	131 (40.9%)	136 (43.7%)	
CCB					0.37
No	356 (69.9%)	164 (64.6%)	227 (70.9%)	212 (68.2%)	
Yes	153 (30.1%)	90 (35.4%)	93 (29.1%)	99 (31.8%)	
ACEI					0.02
No	396 (77.8%)	185 (72.8%)	244 (76.3%)	259 (83.3%)	
Yes	113 (22.2%)	69 (27.2%)	76 (23.8%)	52 (16.7%)	
ARB					0.18
No	401 (78.8%)	193 (76.0%)	246 (76.9%)	224 (72.0%)	
Yes	108 (21.2%)	61 (24.0%)	74 (23.1%)	87 (28.0%)	
Statin					< 0.01
No	281 (55.2%)	160 (63.0%)	222 (69.4%)	234 (75.2%)	
Yes	228 (44.8%)	94 (37.0%)	98 (30.6%)	77 (24.8%)	
Fibrate					0.05
No	472 (92.7%)	229 (90.2%)	306 (95.6%)	294 (94.5%)	
Yes	37 (7.3%)	25 (9.8%)	14 (4.4%)	17 (5.5%)	

*DM alone* diabetes alone, *CKD alone* chronic kidney disease alone, *DM and CKD* both DM and CKD, *previous MI* history of previous myocardial infarction, *CABG history* history of coronary artery bypass graft, *P2Y12 inhibitor* P2Y12 receptor inhibitor of platelets, *BB* beta-blockers, *CCB* calcium channel blocker, *ACEI* angiotensin-converting enzyme inhibitor, *ARB* angiotensin receptor blocker

**Fig. 1:**
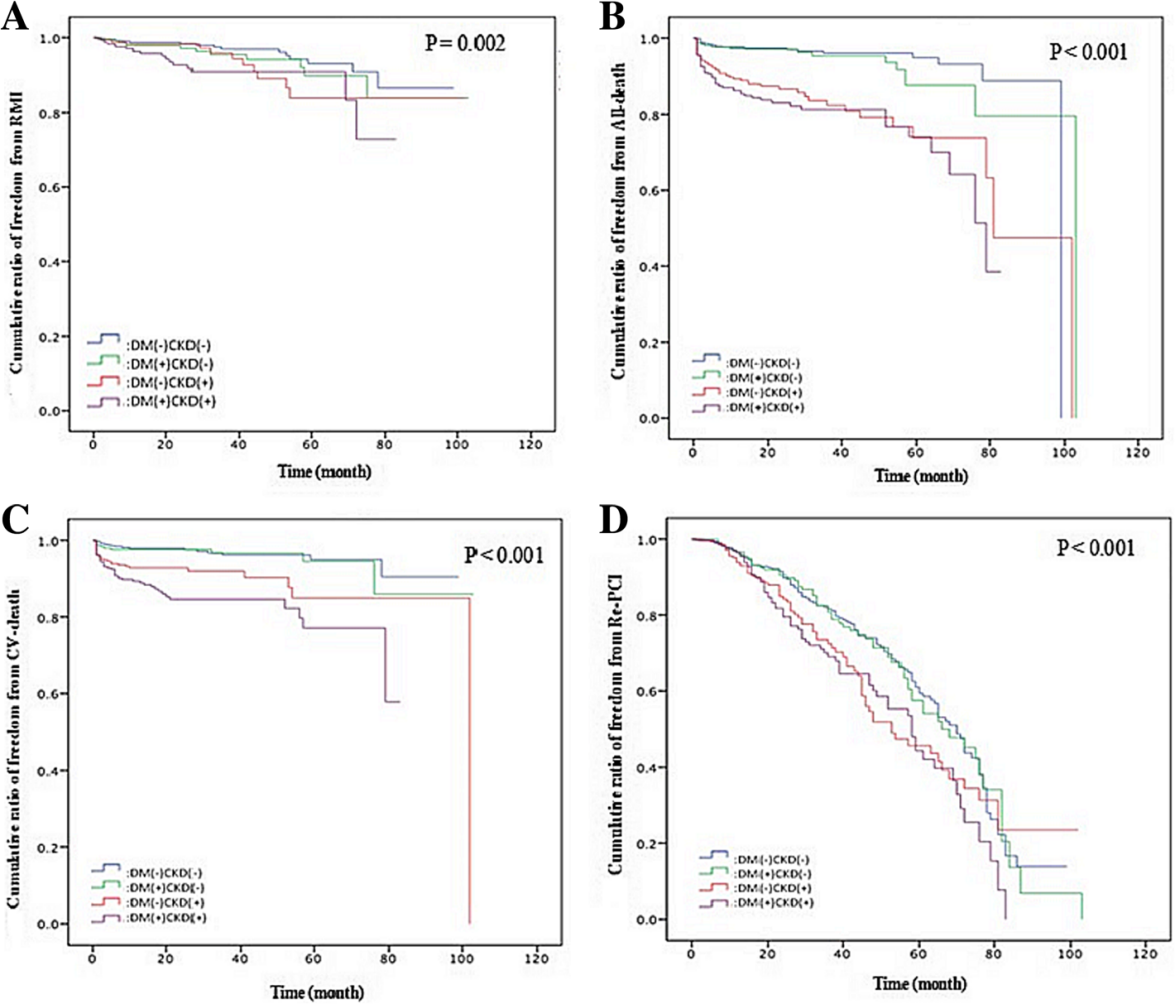
**a** Cumulative ratio of freedom from recurrent MI among the 4 groups (*P* = 0.002). **b** Cumulative ratio of freedom from all death among the 4 groups (*P* < 0.001). **c** Cumulative ratio of freedom from CV death among the 4 groups (*P* < 0.001). **d** Cumulative ratio of freedom from Re-PCI among the 4 groups (*P* < 0.001)

The angiographic findings and clinical outcomes are shown in Table 3. From the angiographic findings, double-vessel and triple-vessel disease were found more frequently in patients with both DM and CKD (*P* < 0.01). There were no differences in invasive strategy among the 4 groups (*P* = 0.34). As for adverse outcomes, patients with both DM and CKD had the highest all-cause mortality and cardiovascular mortality rate (both *P* < 0.01); however, there was no difference in MI or repeated PCI rate among the 4 groups (*P* = 0.09 and *P* = 0.32, respectively). Figure 1 reveals the cumulative rate of freedom from MI, cardiovascular death, all-cause mortality, and repeated PCI among the 4 groups. Freedom from MI, all-cause mortality, CV death, and repeated PCI were lowest in the combined DM and CKD group (*P* = 0.002, *P* < 0.001, *P* < 0.001, and *P* < 0.001, respectively).

**Table 3:** Angiographic Findings and Outcomes

Variable	Study Group				*P* value
	Control (N = 509)	DM alone (N = 254)	CKD alone (N = 320)	DM and CKD(N = 311)	
Follow-up time (months)	31.7 ± 22.6	31.1 ± 22.4	23.8 ± 19.7	23.0 ± 19.2	0.08
Number of diseased vessel					< 0.01*
Single-vessel disease	297 (58.4%)	120 (47.3%)	146 (45.6%)	106 (34.1%)	
Double-vessel disease	138 (27.1%)	75 (29.5%)	90 (28.1%)	112 (36.0%)	
Triple-vessel disease	74 (14.5%)	59 (23.2%)	84 (26.3%)	93 (29.9%)	
Mean treated vessels ^a^	1.2 ± 0.4	1.3 ± 0.5	1.3 ± 0.5	1.3 ± 0.5	< 0.01*
Mean treated lesions	1.4 ± 0.7	1.5 ± 0.8	1.5 ± 0.8	1.6 ± 0.9	0.07
Type of intervention					0.34
Balloon angioplasty (Yes)	163 (32.0%)	72 (28.4%)	109 (34.1%)	109 (35.1%)	
BMS deployment (Yes)	210 (41.3%)	87 (34.3%)	150 (46.9%)	130 (41.8%)	
DES deployment (Yes)	183 (36.0%)	120 (47.2%)	106 (33.1%)	116 (37.3%)	
Lesion location					0.31
LAD	358 (70.3%)	182 (71.7%)	236 (73.8%)	249 (80.0%)	
Lcx	223 (43.8%)	125 (49.2%)	160 (50.0%)	197 (63.3%)	
RCA	218 (42.8%)	125 (49.2%)	186 (58.1%)	182 (58.5%)	
SYNTAX score	9.8 ± 7.2	11.8 ± 8.3	11.0 ± 7.7	11.2 ± 8.1	0.12
LVEF	0.61 ± 0.12	0.59 ± 0.14	0.58 ± 0.14	0.55 ± 0.15	< 0.01*
MI					0.19
Yes	16 (3.1%)	13 (5.1%)	12 (3.8%)	19 (6.1%)	
No	493 (96.9%)	241 (94.9%)	308 (96.2%)	292 (93.9%)	
CV death					< 0.01*
Yes	16 (3.1%)	9 (3.5%)	27 (8.4%)	44 (14.2%)	
No	493 (96.9%)	245 (96.5%)	293 (91.6%)	267 (85.8%)	
All-cause death					< 0.01*
Yes	20 (3.9%)	15 (5.9%)	49 (15.3%)	57 (18.3%)	
No	489 (96.1%)	239 (94.1%)	271 (84.7%)	254 (81.7%)	
Re-PCI					0.87
Yes	127 (25.0%)	63 (24.8%)	72 (22.5%)	74 (23.8%)	
No	382 (75.1%)	191 (75.2%)	248 (77.5%)	237 (76.2%)	

*BMS* bare metal stent, *DES* drug-eluting stent, *LAD* left anterior descending artery, *Lcx* left circumflex artery, *RCA* right coronary artery, *SYNTAX score* Synergy between Percutaneous Coronary Intervention with Taxus and Cardiac Surgery score, *LVEF* left ventricular ejection fraction, *MI* myocardial infarction, *Re-PCI* repeated percutaneous coronary intervention

^*^: significant

^a^ : control vs DM alone: *P*=0.0059, control vs CKD alone: *P* = 0.0027, control vs DM and CKD: *P* = 0.0089, DM vs CKD: *P* = 0.7603, DM vs DM and CKD: *P* = 0.7070, CKD vs DM and CKD: *P* = 0.4160

An outcome analysis and outcome predictors from the Cox proportion hazard model for MI, all-cause mortality, CV death, and repeated PCI are listed in Table 4. Patients with both DM and CKD carried the highest risk, as compared with the control group in terms of MI, CV death, all-cause mortality, and repeated PCI (hazard ratio [HR]: 2.43, 3.25, 3.58, and 1.79, respectively; all *P* < 0.01). Based on the results from the Cox proportional hazard model, we found that previous MI and Syntax scores were predictors of MI (HR: 3.14 and 1.03, respectively), and statin use could reduce the risk of MI (HR: 0.46). Age, previous MI, stroke history, and Syntax score were predictors of all-cause death (HR: 1.04, 3.95, 1.97, and 1.03, respectively), beta blockers (BB) and statin use could reduce the risk (HR: 0.61 and 0.37, respectively). Previous MI, P2Y12 inhibitor use, and Syntax score were all predictors for CV death (HR: 4.29, 2.33, and 1.03, respectively); BB, ACEI, and statin use could reduce the risk of CV death (HR:0.53, 0.42, and 0.41, respectively). Finally, smoking and BB use were associated with repeated PCI (HR:1.57 and 1.40, respectively).

**Table 4:** Significant Outcome Predictors in a Cox proportion hazard model for recurrent MI,All Death, CV Death, and Repeated PCI

Variable	RMI^a^	All-cause death^b^	CV-death^c^	Repeated PCI^d^
	HR^a^ (95% CI)	HR^a^ (95% CI)	HR^a^ (95% CI)	HR^a^ (95% CI)
Group				
Control	1.00 (Reference)	1.00 (Reference)	1.00 (Reference)	1.00 (Reference)
DM alone	1.43 (0.67–3.06)	1.18 (0.59–2.36)	0.91 (0.40–2.09)	1.11 (0.82–1.52)
CKD alone	1.47 (0.71–3.08)	2.04 (1.15–3.63)^*^	2.13 (1.13–4.01)^*^	1.47 (1.09–1.97)^*^
DM and CKD	2.43 (1.23–4.08)^*^	3.25 (1.89–5.59)^**^	3.58 (1.97–6.49)^**^	1.79 (1.33–2.41)^**^
Age	-	1.04 (1.02–1.05)^**^	-	-
Smoking	-	-	-	1.57 (1.25–1.96)^**^
Previous MI	3.14 (1.82–5.40)^**^	3.95 (2.72–5.74)^**^	4.29 (2.71–6.79)^**^	-
Stroke	1.88 (0.75–4.74)	1.97 (1.16–3.36)^*^	1.72 (0.91–3.25)	-
Diuretics	-	-	1.35 (0.85–2.15)	-
DES	-	-	-	-
Asprin		-	1.33 (0.66–2.69)	-
P2Y12 inh		-	2.33 (1.01–5.39)^*^	-
BB	-	0.61 (0.43–0.88)^**^	0.53 (0.34–0.83)^**^	1.40 (1.23–1.74)^**^
CCB	-	-	-	-
ACEI	-	-	0.42 (0.25–0.73)^**^	-
ARB	-	-	-	-
Statin	0.46 (0.26–0.84)^*^	0.37 (0.23–0.58)^**^	0.41 (0.24–0.69)^**^	-
Syntax	1.03 (1.01–1.06)^*^	1.03 (1.01–1.04)^**^	1.03 (1.01–1.05)^**^	1.00 (0.98–1.01)

*DM alone* diabetes alone, *CKD alone* chronic kidney disease alone, estimated glomerular filtration rate < 60 mL/min; *Previous MI* history of previous myocardial infarction, *DES* drug-eluting stent, *P2Y12 inh* P2Y12 receptor inhibitor of platelets, *BB* beta-blockers, *CCB* calcium channel blocker, *ACEI* angiotensin-converting enzyme inhibitor, *ARB* angiotensin receptor blocker, *Syntax score* Synergy between Percutaneous Coronary Intervention with Taxus and Cardiac Surgery score

^*^ *P* < 0.05, ^**^ *P* < 0.01

^a^RMI model: y = βdummyDH1 + βdummyDH2 + βdummyDH3 + βMI + βstroke + βstatin + βsyntax

^b^All-death model: y = βdummyDH1 + βdummyDH2 + βdummyDH3 + βage + βCKD + βMI + βstroke + βbetab + βstatin + βsyntax

^c^CV-death model: y = βdummyDH1 + βdummyDH2 + βdummyDH3 + βMI + βstroke + βdiuretics + βbetab + βACEI + βstatin + βsyntax

^d^Repeated PCI model: y = βdummyDH1 + βdummyDH2 + βdummyDH3 + βMI + βsmoking + βbetab + βsyntax

Table 5 lists the incidence of adverse outcomes according to the different CKD stages. We found that patients with advanced-stage CKD (stage 4 and 5) had higher cardiovascular mortality and all-cause mortality than those with early-stage CKD (both *P* < 0.001). However, there was no difference in terms of recurrent MI or repeated PCI (*P* = 0.06 and *P* = 0.20, respectively).

**Table 5:** Incidence of Adverse Outcomes with Different CKD Stages According to Estimated Glomerular Filtration Rate

Outcome	CKD Stage					*P* value
	1	2	3	4	5	
MI						
Yes	99 (32.5%)	143 (31.8%)	147 (35.0%)	50 (45.9%)	43 (39.1%)	0.0552
No	206 (67.5%)	306 (68.2%)	273 (65.0%)	59 (54.1%)	67 (60.9%)	
CV death						
Yes	11 (3.6%)	14 (3.1%)	32 (7.6%)	21 (19.3%)	18 (16.4%)	<0.0001
No	294 (96.4%)	436 (96.9%)	388 (92.4%)	88 (80.7%)	92 (83.6%)	
All-cause death						
Yes	16 (5.3%)	19 (11.4%)	48 (11.4%)	32 (29.4%)	26 (23.6%)	<0.0001
No	289 (94.7%)	431 (95.8%)	372 (88.6%)	77 (70.6%)	84 (76.4%)	
Re-PCI						
Yes	66 (21.6%)	124 (27.6%)	100 (23.8%)	20 (18.4%)	26 (23.6%)	0.2029
No	239 (78.4%)	326 (72.4%)	320 (76.2%)	89 (81.6%)	84 (76.4%)	

## Discussion

In patients with CAD undergoing PCI, we found that the highest rate of all-cause mortality, CV mortality, myocardial infarction, and repeated PCI occurred in patients with both DM and CKD, as compared with patients without DM and CKD, patients with DM alone, and patients with CKD alone. Patients with CKD alone had the second highest risk of all-cause mortality, cardiovascular mortality, and repeated PCI. As for the outcome analysis, we found that smoking and BB use was associated with repeated PCI procedures. Previous MI and Syntax scores were predictors of MI, and statin use could reduce the risk of MI. Age, previous MI, stroke history, and Syntax score were predictors of all-cause death, and BB and statin use could reduce the risk. Previous MI, P2Y12 inhibitor use, and Syntax score were all predictors of CV death, and BB, ACEI, and statin use could reduce the risk of CV death.

In our study, patients with CKD alone, along with patients with both DM and CKD, were older and had a lower BMI; this is compatible with a previous study [17]. Patients with both DM and CKD had a more elevated serum creatinine level than patients with CKD alone (*P* < 0.001), implying that DM had an adverse impact on renal function in CKD patients. Secondarily, patients with both DM and CKD had the highest CSP and lowest CDP, thus they had the most elevated central pulse pressure (CPP). Elevated CPP is strongly associated with adverse cardiovascular outcomes in patients with hypertension patients and in patients after undergoing a PCI procedure in a previous study [18, 19]. Elevated CPP in patients with DM and CKD may reflect that they had the most advanced arterial stiffness among the 4 groups.

Patients with both DM and CKD had a higher prevalence rate of hypertension than patients with isolated DM or isolated CKD (both *P* < 0.05), suggesting that DM and CKD had an additive effect on the acceleration of hypertension. In contrast, compared with patients with DM alone or CKD alone, patients with both DM and CKD used statins less frequently as they had the lowest serum cholesterol and LDL-C levels. Similarly, ACEI usage was also the least since patients with both DM and CKD had the poorest renal function and concern of increased hazard of hyperkalemia. When a patient has an LDL of <70 mg/dL, statin use has been found to improve cardiovascular outcomes in CAD patients after ACS [20]; however, whether statin under-usage led to the poorest outcome in patients with both DM and CKD in this study remains to be clarified.

Given for lesion location, intervention type such as balloon angioplasty, bare metal stent deployment, or drug-eluting stent deployment, there were no difference. However, patients with both DM and CKD had the highest prevalence of double-vessel disease and triple-vessel disease; they also had the lowest LVEF. Compared with patients with DM alone, patients with both DM and CKD had a significantly higher risk of developing multi-vessel disease (*P* = 0.006); when compared with patients with CKD alone, patients with both DM and CKD also had a significantly higher risk of developing multi-vessel disease (*P* = 0.01). Combined DM and CKD seemed to have an additive effect on the progression of atherosclerosis and development of multi-vessel disease than patients with isolated DM or isolated CKD. This is in contrast to a previous study that they observed patients with both DM and hypertension did not have a significant risk of developing multi-vessel disease, as compared with patients with DM alone [21]. However, in terms of treated numbers of the vessels and lesions, no difference existed among patients with DM alone, CKD alone, or both DM and CKD (*P* = NS); thus, dominance of multi-vessel disease and poor left ventricular function in patients with DM and CKD may affect long-term outcomes in the present study. Beyond conventional risk factors for the development of CAD, other important prognostic indicators to assess in CAD patients include LVEF and number of diseased vessels [22].

In our study, patients with DM and CKD had the highest rate of all-cause mortality and CV mortality. This may be due to patients with both DM and CKD had a higher rate of multi-vessel disease, along with a poorer LVEF, less statin use, and less ACEI use. On the contrary, while comparing patients with DM alone and CKD alone, patients with CKD alone had an increased rate of all-cause mortality and CV mortality than patients with DM alone (*P* < 0.001 and *P* = 0.02, respectively). However, there were no significant differences between the 2 groups regarding the number of diseased vessels, lesion complexity, LVEF, and medications. In this study, patients with CKD seemed had a poorer outcome than patient with DM. As been stated, a combination of insulin resistance and endothelial dysfunction leads to the progression of atherosclerosis in patients with DM and patients with CKD [23, 24]; a larger scale clinical trial is necessitated to determine whether patients with CKD have more advanced insulin resistance and endovascular dysfunction than patients with DM.

In conclusion, we found that patients with DM and CKD had the highest mortality after PCI than isolated DM or isolated CKD.; DM and CKD had an additive effect on long-term risks. In terms of adverse outcomes, patients with CKD seemed more hazardous than patients with DM.

## Conclusions

DM and CKD had additive effect on adverse long-term outcomes in patients with.

CAD after receiving PCI. In patients receiving PCI, CKD was a more hazardous outcome predictor than DM.

### Study limitations

First, were did not fully evaluated the intensity of medication, such as tight blood glucose control or blood pressure control. Second, data entry bias may exist; functional evaluations of the atherosclerotic lesions such as fraction flow reserve (FFR) measurement, were not used that may have had an impact on the decision of index PCI enrollment; it has been reported that DM and CKD were independent predictors for FFR measurement. Besides, positive findings of FFR were lower in patients with CKD whereas index of microcirculatory resistance (IMR) was higher [25].Third, since the number of patients in the DM alone group was fewer than that of the other groups, that may have affected the power of this study. Fourth, since DES implantation had a lower adverse cardiac events rate in patients with diabetes or CKD than BMS implantation, patient selection bias may exist in this study.

## Abbreviations

ACEI: Angiotensin-converting enzyme inhibitor
ARB: Angiotensin receptor blocker.
BB: Beta-blockers
BMI: Body mass index
BMS: Bare-metal stent
CABG: Coronary artery bypass graft
CAD: Coronary artery disease
CAP: Central aortic pressure
CCB: Calcium channel blockers
CDP: Central aortic diastolic pressure
CKD: Chronic kidney disease
CSP: Central aortic systolic pressure
CV Mortality: cardiovascular mortality
DES: Drug-eluting stent
eGFR: Estimated glomerular filtration rate
FFR: Fraction flow reserve
HbA1c: Hemoglobin A1C
HDL-C: High-density lipoprotein-cholesterol
LAD: Left anterior descending artery
Lcx: Left circumflex artery
LDL-C: Low-density lipoprotein- cholesterol
LVEF: Left ventricular ejection fraction
MI: Myocardial infarction
OPD: Outpatient department
PCI: Percutaneous coronary intervention
RCA: Right coronary artery
SYNTAX score: Synergy between percutaneous coronary intervention with taxus and cardiac surgery score
TG: Triglyceride
